# p38 Signaling and Receptor Recycling Events in a Microfluidic Endothelial Cell Adhesion Assay

**DOI:** 10.1371/journal.pone.0065828

**Published:** 2013-06-07

**Authors:** Dwayne A. L. Vickers, Emma J. Chory, Megan C. Harless, Shashi K. Murthy

**Affiliations:** 1 Department of Chemical Engineering, Northeastern University, Boston, Massachusetts, United States of America; 2 Barnett Institute of Chemical and Biological Analysis, Northeastern University, Boston, Massachusetts, United States of America; University of Nebraska Medical Center, United States of America

## Abstract

Adhesion-based microfluidic cell separation has proven to be very useful in applications ranging from cancer diagnostics to tissue engineering. This process involves functionalizing microchannel surfaces with a capture molecule. High specificity and purity capture can be achieved using this method. Despite these advances, little is known about the mechanisms that govern cell capture within these devices and their relationships to basic process parameters such as fluid shear stress and the presence of soluble factors. This work examines how the adhesion of human endothelial cells (ECs) is influenced by a soluble tetrapeptide, Arg-Glu-Asp-Val (REDV) and fluidic shear stress. The ability of these ECs to bind within microchannels coated with REDV is shown to be governed by shear- and soluble-factor mediated changes in p38 mitogen-activated protein kinase expression together with recycling of adhesion receptors from the endosome.

## Introduction

Endothelial cells (ECs) line the blood vessel walls and serve as an interface for blood flow [1], [2]. Due to their unique location they are constantly exposed to a multitude of mechanical forces. The hemodynamic force from the luminal blood, together with adhesive forces between cell surface anchoring proteins (integrins) and the basement membrane (basal lamina) contribute to a complex set of mechanical signals that are known to regulate vascular function through multiple, mechanotransduction-related signaling pathways [3], [4]. Based on their important role in the cardiovascular system attention has been focussed on isolating both immature and mature ECs from heterogeneous starting material for applications such as tissue engineering [5], [6], [7].

Microfluidic cell separation has emerged as an attractive alternative to MACS and FACS due to the small sample volume requirement and the ability to capture rare cell populations [8], [9]. More specifically, adhesion-based cell separation has proven to be very useful in a wide range of applications, ranging from cancer diagnostics to tissue engineering because it eliminates the need for sample pre-processing to bind fluorescent or magnetic tags [8], [9], [10], [11]. This approach involves functionalizing microfluidic channels with molecules that bind to one or more cell types that are captured from a flowing stream. Various research groups have demonstrated the utility of this approach in terms of capturing the target cells [5], [12], [13], [14]. Little is known, however, about the effect that such capture platforms may have on cells during the isolation process, particularly when dealing with shear-sensitive cells, such as ECs and stem cells. At present, it is generally assumed that cells are quiescent during the separation process and no change occurs within the cells that would substantially affect affinity. This assumption enables the design of microfluidic capture systems based on simple profiles of cell adhesion as function of shear stress [9].

In previous work by our group, a series of experiments demonstrated that cell adhesiveness can indeed be altered by exposure to shear and soluble molecules [15]. These studies involved blocking surface receptors of human umbilical vein endothelial cells (HUVECs) with the ligand Arg-Glu-Asp-Val (REDV) followed by flow within REDV functionalized microchannnels. The objective of this study is to relate such changes to specific intracellular mechanisms and processes.

## Materials and Methods

### Materials

Ethanol (200 proof), cover slips (35×60 mm, no. 1), microcentrifuge tubes, and cell culture flasks were purchased from Fisher Scientific (Fair Lawn, NJ). 3-Mercaptopropyl trimethoxysilane was obtained from Gelest Inc. (Morrisville, PA) and the coupling agent GMBS (N-γ-maleimidobutyryloxy succinimide ester) was obtained from Pierce Biotechnology (Rockford, IL). SU-8-50 photoresist and developer were obtained from MicroChem (Newton, MA); silicone elastomer and curing agent were obtained from Dow Corning (Midland, MI). Phosphate buffered saline (PBS; 1×, without calcium or magnesium) was purchased from Mediatech (Herndon, VA). HUVECs, singlequot kit supplements and growth factors, HEPES buffered saline solution, trypsin neutralizing solution, and 0.25% trypsin/EDTA solution were purchased from Lonza (Walkersville, MD). The peptide REDV, along with REDV conjugated to fluorescein isothiocyanate (FITC) were purchased from American Peptide (Sunnyvale, CA). Quantum FITC-5 MESF kits were purchased from Bangs Laboratory (Fishers, IN). The inhibitors BAPTA, PD98059, SP600125 and SB202190 were purchased from Invitrogen (Grand Island, NY). The activated anti-integrin β_1_ antibody was purchased from Millipore (Billerica, MA, Cat# FCMAB389F).

### Microchannel Fabrication

Microchannels were fabricated using standard soft lithography techniques [16], [17]. Negative masters for device fabrication were manufactured at the George J. Kostas Nanoscale Technology and Manufacturing Research Center at Northeastern University. For master fabrication, 2-dimensional projections of each device type were drawn using AutoCAD and printed at high resolution on transparencies (FineLine Imaging, Colorado Springs, CO). The photomask was then used to generate a negative master. A silicon wafer was coated with SU 8-50 photoresist to a thickness of approximately 70 µm and exposed to ultraviolet light (365 nm, 11 mW/cm^3^) with the transparency overlaid using a Quintel 2001 mask aligner. Following curing, the unexposed photoresist was removed using SU 8 developer, and the feature height verified using a Dektak surface profiler (Veeco Instruments, Santa Barbara, CA). A straight channel device with dimensions of 1×50×0.07 mm (width×length×height) was used for all microfluidic flow experiments. Polydimethysiloxane (PDMS) replicas were generated using silicone elastomer and curing agents in the ratio of 10∶1 (w/w). This mixture was poured onto the negative master and allowed to degas, then cured at 65°C for 2 h. PDMS replicas were peeled off the wafers prior to punching inlet and outlet holes with a 19-gauge blunt-nose needle. The replicas and glass slides were exposed to an oxygen plasma (100 mW with 8% oxygen for 30 s) in a PX-250 plasma chamber (March Instruments, Concord, MA) and then immediately placed in contact with each other. The irreversible bonding between PDMS and glass was completed by baking for 5 min at 65°C. Surface functionalization was performed immediately after the baking step.

### Surface Modification

A three-step protocol was employed to bind REDV to the microchannel surface. First, 4% (v/v) solution of 3-mercaptopropyl trimethoxysilane in ethanol was prepared under a nitrogen atmosphere and flowed into each channel. This solution was left to react undisturbed for 30 min. The unreacted silane was flushed out with ethanol and followed by injection of 0.28% GMBS in ethanol solution flushed into the device. The GMBS was left to react for 15 min. Following this reaction period, ethanol was used to flush out the unreacted GMBS, followed by a rinse with PBS. A solution of 0.1 mg/mL of REDV in PBS was then injected into each microchannel. Following a 30 min period, the devices were flushed with PBS and either used directly in experiments or stored at 4°C.

### Cell Culture

HUVECs obtained from Lonza were cultured in 150 cm^2^ tissue culture flasks at 37°C in a humidified atmosphere with 5% CO_2_ and 95% air in vendor-supplied culture medium (Lonza, Walkersville, MD). The medium used was Endothelial Basal Medium-2 (EBM-2) with the following added supplements: hydrocortisone, hFGF-B, 0.39%; VEGF, 0.097%; R3-IGF-1, 0.097%; ascorbic acid, 0.097%; heparin, 0.097%; FBS, 1.97%; hEGF, 0.097%; and GA-1000, 0.097% (v/v). HUVECs were grown to 80% confluence and isolated for experiments by trypsinization using a 0.25% trypsin-EDTA solution and used for 3 passages only. Cell suspensions were centrifuged at 190× *g* and then resuspended in serum-free medium (EBM-2) at a concentration of 5×10^5^ cells/mL for all flow experiments.

### Straight Channel Shear Flow Experiments

HUVECs were incubated in various concentrations of REDV (0, 50, 75 and 100 µg/mL) in serum free medium in closed centrifuge tubes. This process was followed by centrifugation and resuspension in serum-free culture medium. A 0.6 mL suspension of cells at a concentration of 1×10^6^ cells/mL was flowed into straight channel devices at a flow rate of 5.5 µL/min for 32 min which results in each cell experiencing shear for a residence time period (volume/volumetric flow rate) of 45 s. This flow rate corresponds to a wall shear stress of 1.1 dyn/cm^2^. Cell adhesion within the devices was measured using a field finder (with 1 mm×1 mm grids) placed under the microfluidic chamber. Cell counts were taken at locations corresponding to ¼, ½, and ¾×the total channel length. As in previous studies of cell adhesion in microchannels by our group, the uniformity of the input cell concentration was carried out via multiple measurements and flow cytometry [5], [13], [16].

### Integrin Activation Experiments

Activated integrin anti-β_1_ (HUTS-4 clone) antibodies were used to confirm the presence of activated β_1_ integrins before and after exposure to soluble REDV and shear stress. First, the total number of activated integrin β_1_ receptors that HUVECs possess were evaluated by incubating the cells at a concentration of 1×10^6^ cells/mL in a 1∶10 dilution of FITC conjugated anti-β_1_ integrin for 30 min. These cells were then centrifuged and resuspended in serum-free media for analysis of the fluorescence intensity in the flow cytometer where the total number of activated β_1_ integrin receptors was measured. The effect of soluble REDV on the activated integrin β_1_ receptors was evaluated by incubating HUVECs in the various concentrations of REDV for 30 min, followed by centrifugation and resuspension in a 1∶10 dilution of FITC conjugated anti-β_1_ integrin at a concentration of 1×10^6^ cells/mL. This suspension was incubated for 30 min followed by centrifugation and resuspension in serum-free medium for fluorescence intensity analysis in a flow cytometer as described below.

The effect of soluble REDV combined with flow on the activated integrin β_1_ receptors was evaluated by incubating HUVECs in the various concentrations of REDV as reported earlier [15]. For these experiments the HUVEC cells were flowed into straight channel devices at a flow rate of 5.5 µL/min (1.1 dyn/cm^2^) for 32 min and the outflow collected and incubated for 30 min in a 1∶10 dilution of FITC conjugated anti-β_1_ integrin. Following incubation the cells were centrifuged and resuspended in serum free medium for fluorescence intensity measurements in the flow cytometer.

### Receptor Number Evaluation Via Fluorescence Intensity

To determine the number of available receptors fluorescence intensity measurements obtained from the flow cytometer were related to fluorescence measurements obtained from standard beads with known levels of fluorescence. Quantum FITC-5 MESF kits comprising six types of microspheres, i.e. specifically five populations having different known levels of FITC and one blank population, were used for this calibration. A standard curve relating fluorescence intensity to standardized MESF values from quantum MESF beads was generated by loading the beads into the flow cytometer (Beckman Coulter Quanta SC) and recording the fluorescence intensity. The number of unoccupied REDV receptors was determined by incubating HUVECs in FITC conjugated REDV for 30 mins followed by analysis with the flow cytometer where the readout is given as fluorescence intensity. The number of receptors was then extracted from the calibration curve developed prior.

### Pathway Inhibition Experiments

For this experiment HUVECs at a concentration of 1×10^6^ cells/mL were first inhibited of calcium, extracellular signal-regulated kinases (ERK ½), Jun amino-terminal kinases (JNK) and p38 MAP kinase by incubating them in 10 µM of BAPTA, 25 µM of PD98059, 10 µM SP600125 and 10 µM of SB202190, respectively, in serum-free medium for 30 min, where each inhibitor concentration was chosen from previous studies in which optimum inhibition was achieved without sacrificing the cell's integrity. Cell morphology did not change after inhibition. The cells were centrifuged and resuspended in serum-free medium and then incubated in the previously mentioned concentrations of REDV. Next, these cells were flowed into straight channel devices and cell adhesion within the devices enumerated as previously described.

### Evaluation of Receptor Number after p38 Inhibition

HUVECs were incubated in 10 µM of SB202190 as previously mentioned. The cells were then centrifuged and resuspended in varying concentrations of REDV for 30 min. This was followed by incubation in 300 µg/mL of REDV-FITC (a saturating concentration level) for 30 min followed by fluorescence intensity measurements to obtain the number of unbound REDV binding receptors.

### Receptor Number Evaluation After p38 Inhibition followed by Soluble REDV Incubation and Shear

HUVEC suspensions were incubated in 10 µM of SB202190 for 30 min and resuspended in the mentioned concentrations of REDV for 30 min. The cell suspension was flowed into REDV functionalized devices and the outflow collected and incubated in 300 µg/mL of REDV-FITC for 30 min. The cell suspension was then centrifuged and resuspended in serum-free medium for fluorescence intensity measurements in the flow cytometer to determine the number of unbound REDV binding receptors.

### Receptor Recycling Experiments

#### Evaluation of Receptor Number in the Presence of Soluble REDV

HUVECs were incubated in 10 µM monensin for 30 min to inhibit receptor recycling (At a 10 µM concentration of monensin receptor recycling has been reported to be completely inhibited [18], [19].). They were then resuspended in the previously mentioned concentrations of REDV and incubated for 30 min. The cells were then resuspended in 300 µg/mL of REDV-FITC for 30 min and centrifuged and resuspended in serum-free media. Next, these cells were loaded into the flow cytometer and the median intensity peak measurements were recorded. The resulting data was utilized to obtain the number of unbound REDV binding receptors.

#### Evaluation of Receptor Number in the Presence of Soluble REDV and Shear

HUVEC suspensions with a concentration of 1×10^6^ cells/mL were incubated in the mentioned concentrations of REDV for 30 min following the recycling inhibition step in. This was followed by flow in REDV functionalized microchannels. The output from this device was collected and incubated in 300 µg/ml of REDV-FITC for 30 min and fluorescence intensity measurements were made in the flow cytometer as described above.

#### Relation of Cell Adhesion to Receptor Recycling

To determine whether receptor recycling played a role in the rapid increase in receptor numbers observed previously, HUVECs were incubated in 10 µM monensin to inhibit recycling. These cells were then centrifuged and resuspended in the mentioned concentrations of REDV. This was followed by flow into straight channel devices to enumerate cell adhesion.

### Statistics and Data Analysis

For each flow condition studied, 5 repetitions of experiments were performed unless otherwise specified. Error bars represent the standard errors of the mean (standard deviation/√*n*, where *n* = 5). One-way analysis of variance (ANOVA) was performed to investigate the relationship between inhibited and uninhibited conditions. This statistical analysis was executed using KaleidaGraph 4.0. A relatively stringent *p* value threshold (≤0.001 for significance) was applied because of the small overall number of cells adhering within the channels (maximum of ∼50 cells/mm^2^) and to allow better discernment of differences between conditions.

## Results and Discussion

**Figure 1:**
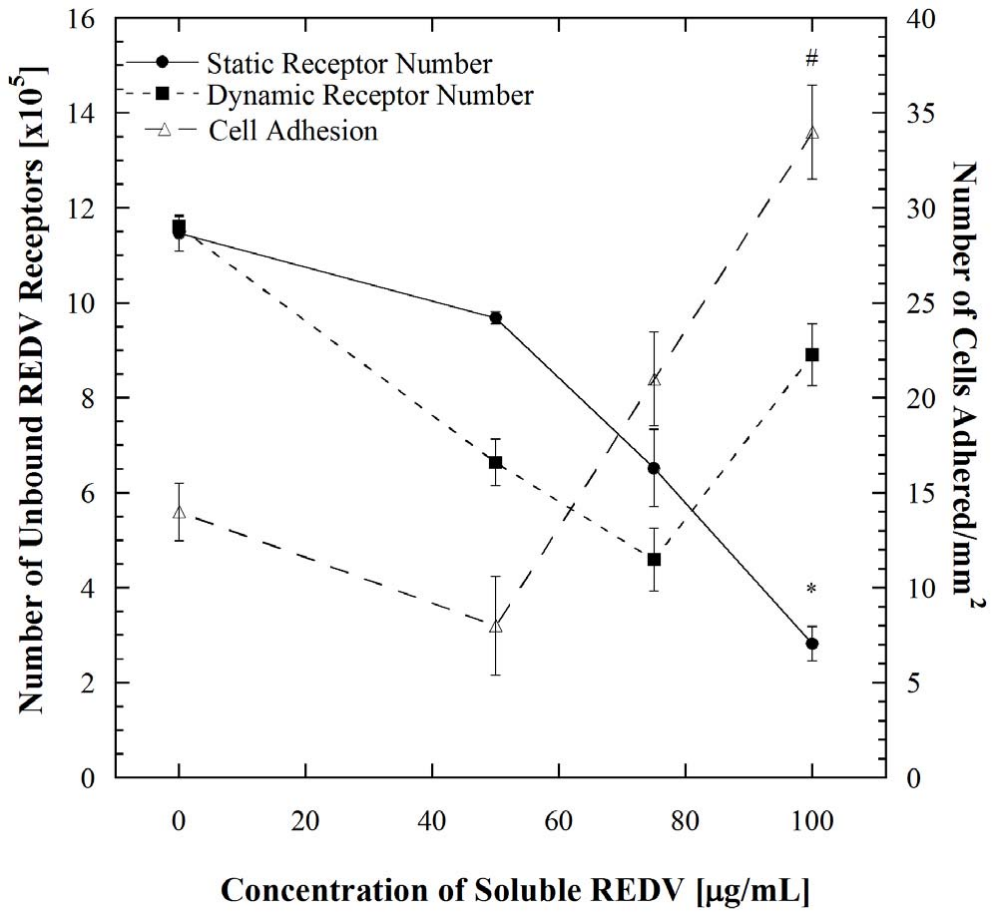
Receptor number analysis of HUVECs before (static) and after flow through microchannels at a shear stress of 1.1 dyn/cm^2^ (dynamic), and cell adhesion as a function of soluble REDV. Error bars denote standard errors for each point based on 3 repetitions for receptor number and 5 repetitions for cell adhesion. # denotes significant difference with *p*<0.001 compared to cell adhesion without REDV pre-incubation and * denotes significant difference with *p*<0.001 compared with the dynamic data point for 100 µg/mL soluble REDV. Adapted from prior study by Vickers and Murthy [15].

This work focuses on understanding changes in the adhesiveness of EC receptors under microfluidic flow. These changes were assessed by first incubating HUVECs in the mentioned REDV concentrations to block all REDV binding receptors followed by flow within REDV functionalized microfluidic channels to evaluate cell adhesion. In previous work from our group REDV was used to capture endothelial cells (ECs) from a heterogeneous population due to the high selectivity that this ligand has for ECs [5], [16]. Consequently, this ligand is used in the blocking studies. Fig. 1. summarizes the findings from a series of REDV blocking experiments that we recently described [15]. In this figure, cell adhesion and REDV binding receptor number are plotted as a function of concentration of soluble REDV. The adhesion aspect of this data shows that incubation in soluble REDV followed by flow of HUVECs through REDV-coated microchannels for ∼45 s residence time causes a decrease in cell adhesion within the microchannels for concentrations of 50 µg/mL soluble REDV and below. When the cells were incubated in soluble REDV solutions of higher concentrations, a counterintuitive increase in cell adhesion was observed. To understand this trend, the number of REDV binding receptors was evaluated for each concentration of incubated soluble REDV to confirm that cell adhesion at the highest concentration of REDV incubation was due to an increase in surface receptor number. This data (solid circles and squares in Fig. 1) compares both static and dynamic conditions. Here, ‘static’ refers to cells that were incubated in REDV solutions of specified concentrations but not flowed through REDV-coated microchannels and ‘dynamic’ refers to cells that were flowed through microchannels following incubation. As shown in Fig. 1, an increase in receptor number is observed when HUVECs are incubated in the highest REDV concentration followed by flow in REDV-coated microchannels. Such an increase is not observed when the cells are not flowed through these microchannels; rather in this case, the available number of receptors decreases monotonically as the cells are incubated with higher concentrations of soluble REDV.

**Figure 2:**
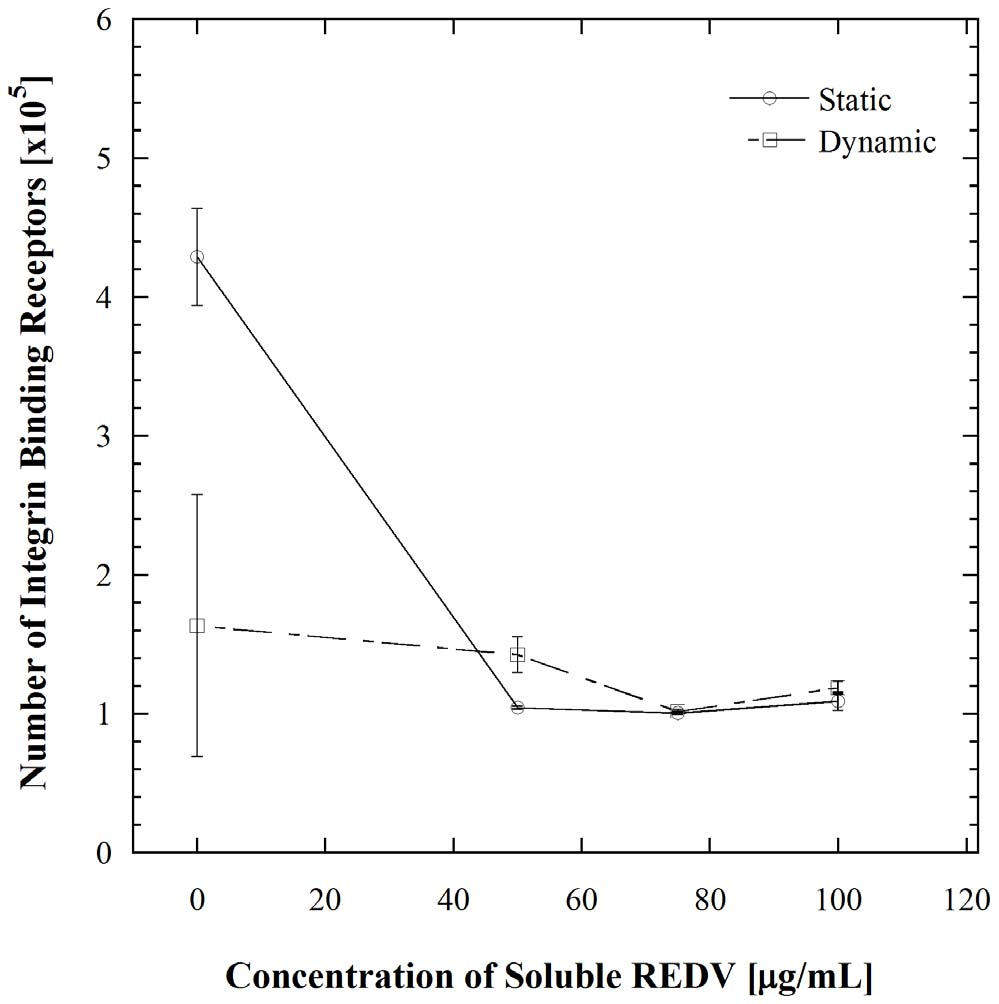
Receptor number analysis of activated β_1_ integrins on HUVECs incubated prior with various concentrations of REDV (static) and after flow through microchannels at a shear stress of 1.1 dyn/cm^2^ (dynamic). Error bars denote standard errors for each point based on 3 repetitions. There is no statistical significance between the static and dynamic cases even at the 0 µg/mL REDV point where there appears to be a difference (*p* = 0.23).

The present work examines several hypotheses to explain the counterintuitive increase in cell adhesion and receptor number which occurs within 45 s of microchannel flow. In our previous work we showed that the interaction between HUVECs and the immobilized REDV is mediated by α_4_ and β_1_ integrins. This was determined by enumerating cell adhesion after incubation in anti- integrin α_4_ and β_1_ blocking antibodies followed by incubation in the mentioned concentrations of REDV [15]. This evaluation, on its own however, does not fully explain the results obtained. Integrins are capable of changing their conformation on the cell surface from a kinetically unfavorable to a favorable one. Such a conformational change could occur in a short time and result in an increase in activated receptors present on the surface [20], [21]. To examine the possibility that exposure of the cells to soluble REDV prior to shear stimulation in the microchannels was activating previously inactive integrin receptors, the number of activated β_1_ integrins was measured at each condition using antibody labeling (Fig. 2). The effect of microchannel flow was then determined by repeating the static experimental steps followed by flow in the microchannel. While there is a difference in the number of activated β_1_ integrins at the condition of no soluble REDV exposure in the static versus dynamic cases, this difference is not significant (*p = *0.23). This insignificant difference between the static and dynamic condition is also reflected at higher levels of REDV pre-incubation (*p*>0.001). This similarity in the levels of β_1_ integrins on the cells surfaces indicates that the counterintuitive increase in cell adhesion shown in Fig. 1 is not a result of activation of previously inactive integrins on the HUVECs surface.

**Figure 3:**
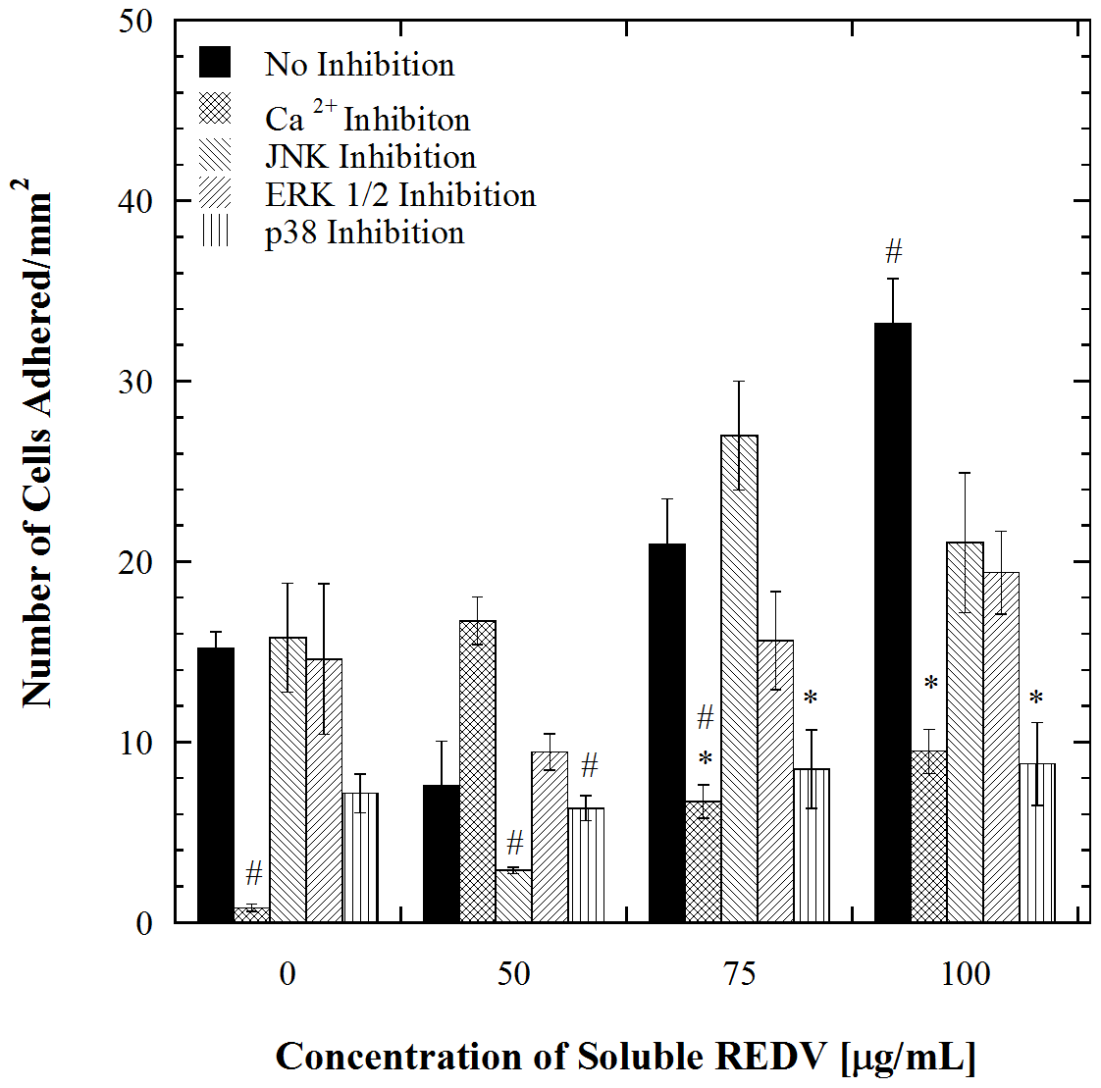
Adhesion of HUVECs on REDV-coated channels at a shear stress of 1.1 dyn/cm^2^ after incubation with the various inhibitors followed by incubation with various concentrations of REDV. Error bars denote standard errors for each point based on 5 repetitions. * denotes significant difference with *p*<0.001 compared to the uninhibited condition for each REDV concentration and # denotes significant difference with *p*<0.001 compared to the no inhibition case at 0 µg/mL REDV.

To examine the role of intracellular signaling, the possibility of calcium transients and mitogen activated protein kinase (MAPK) based activity was probed next. This series of experiments entailed systematically inhibiting each pathway prior to incubation in soluble REDV and microchannel flow. Prior to performing these studies the concentration of inhibitor required to completely suppress a given pathway was determined by comparing inhibition dose responses from the literature. It was determined from these comparisons that the optimum concentration to achieve effective inhibition of calcium, extracellular signal-regulated kinases (ERK ½), Jun amino-terminal kinases (JNK) and p38 MAP kinase are 10 µM of BAPTA [22], 25 µM of PD98059 [23], [24], 10 µM SP600125 [25] and 10 µM of SB202190 [26], [27] respectively. These doses were found to be effective at inhibition while not destroying the cells integrity, furthermore these doses have also been used in other studies for inhibiting HUVECs [28], [29], [30], [31]. Consequently these concentrations are ideal for the inhibition studies that follow. The associated cell adhesion measurements for the inhibition studies follow and are shown in Fig. 3. Here, significantly lower cell adhesion relative to the uninhibited case at the highest concentration of soluble REDV is indicative that the inhibited pathway affects surface receptor numbers. All inhibited conditions within a given group are compared to the uninhibited condition which is the cell adhesion data represented in Fig. 1. Calcium transient was considered first for inhibition because it is one of the most rapid response mechanisms that ECs have to shear stress [32]. The inhibition of calcium transients caused cell adhesion to decrease for all soluble REDV concentrations examined (Fig. 3) except at the 50 µg/mL REDV concentration. This decrease in adhesion at all concentrations of REDV except at the 50 µg/mL makes it difficult to deduce the role of calcium transient in the rapid receptor number increase observed. Furthermore a comparison between the non-inhibited and Ca^2+^ inhibited conditions after no REDV incubation shows a significant cell adhesion decrease for the inhibited cells (*p*<0.001). From this significant decrease it can be inferred that calcium transient inhibition interferes with the cell's normal function. Incubation of the Ca^2+^ inhibited cells in 50 µg/mL of REDV however rescues the initial response observed at 0 µg/mL of soluble REDV. This is evident by the statistical insignificance observed between the non-inhibited cells not incubated in REDV and the Ca^2+^ inhibited cells pre-incubated in 50 µg/mL of REDV (*p* = 0.074). After REDV rescues the effect of Ca^2+^ inhibition, there is no significant difference between the inhibited cells incubated in 100 µg/mL and the original case where the non-inhibited cells are not incubated in REDV (*p*>0.001). Due to the inconsistencies in the cell adhesion trend after Ca^2+^ inhibition along with REDV presence changing the effect of calcium transient inhibition it is difficult to construe the role of calcium transients in the REDV-shear mediated receptor changes. A clear conclusion about calcium transient's role would be possible if there was a reduction in cell adhesion only for the cells incubated in 75 and 100 µg/mL of REDV after calcium transient inhibition. However, such behavior was not observed, suggesting that other cellular processes may be inhibited along with calcium transient. Endothelial cytosolic calcium is associated with multiple shear-associated physiological and biochemical changes and hence it is not surprising that its inhibition brought about the effects observed [33]

**Figure 4:**
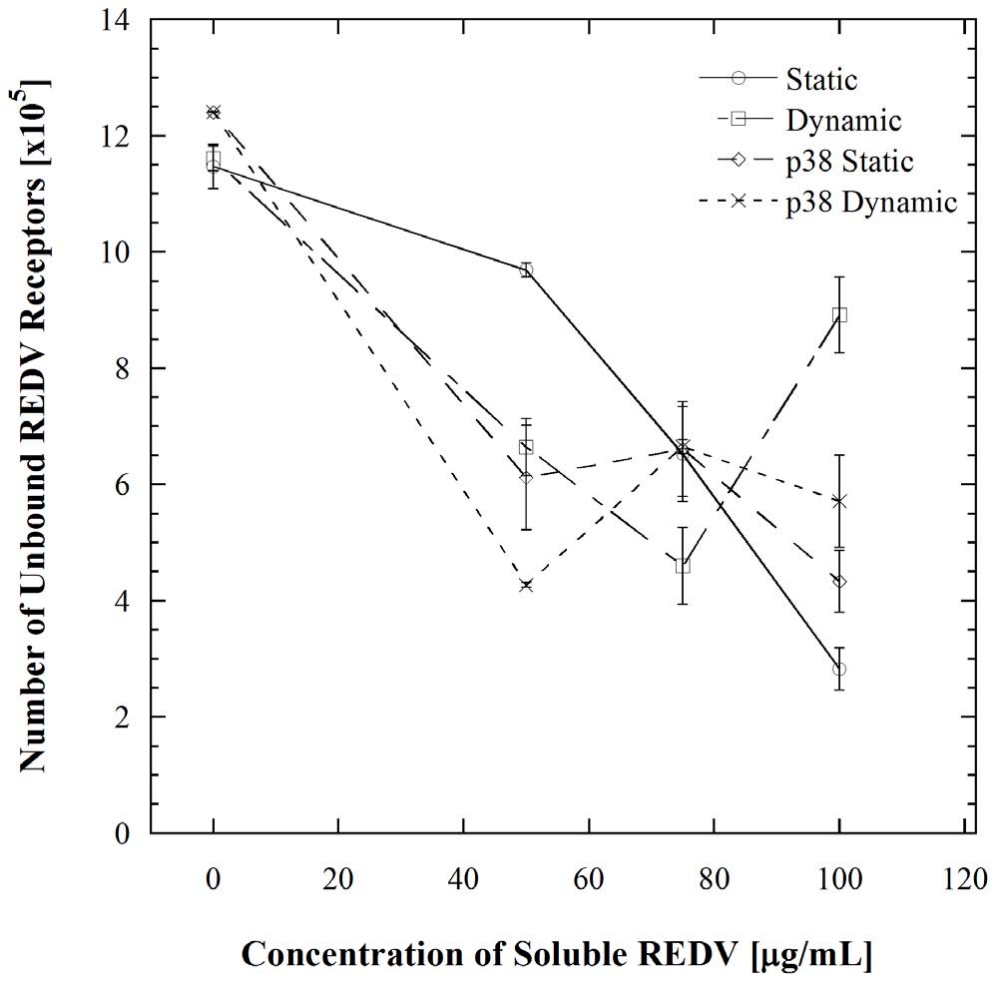
Receptor number analysis of HUVECs where p38 was inhibited before (static) and after flow through microchannels at a shear stress of 1.1 dyn/cm^2^ (dynamic). Upon pre-incubation in 100 µg/mL of REDV without p38 inhibition, the number of receptors in the dynamic case is significantly higher than in the static case (*p<0.001*). With p38 inhibition, the static and dynamic cases are not significantly different (*p* = 0.907). Statistical comparisons of the 50, 75, and 100 µg/mL points within each condition showed no significant difference (*p>0.001*).

Signaling associated with mitogen activated protein kinase (MAPK) is one of the downstream processes that the inhibition of calcium transients could potentially have hindered [34], hence we systematically inhibited the MAPKs that are affected by shear stress in a short period of time. MAPKs belong to a class of signal transduction proteins that are capable of transmitting extracellular signals to the cytoplasmic and nuclear pathways [35]. There are five groups of MAPKs that have been characterized in mammalian cells. These include extracellular signal-regulated kinases (ERKs) 1 and 2 (ERK1/2), c-Jun amino-terminal kinases (JNKs), and p38. When JNK is inhibited, the adhesion trend at all concentrations of soluble REDV is not statistically different from that observed with the uninhibited cells (*p*>0.001). This suggests that JNK is not responsible for the rapid increase in receptors observed in Fig. 1. The inhibition of ERK 1/2 showed a similar trend where there is no significant difference between the uninhibited and inhibited cases at all concentrations of REDV studied (*p*>0.001 in all instances). This suggests that ERK also does not cause the rapid increase in surface receptors. The inhibition of p38 showed a signature trend of what would be expected if a pathway had indeed caused the REDV incubation plus shear mediated cell adhesion increase. The inhibition of this pathway followed by REDV incubation and then microchannel flow resulted in a similar trend to that observed in the uninhibited case for 0 µg/mL and 50 µg/mL of incubated REDV. When the HUVECs are incubated in 75 and 100 µg/mL of REDV, a significant suppression is observed relative to the uninhibited condition for each concentration (*p*<0.001) indicating that the p38 pathway is critical for REDV-shear mediated receptor number increase. This observation is interesting considering that earlier reports of p38 activation show changes occurring in a time-scale of around 10 mins [36]. Our observation therefore suggests that there are other mechanisms that are allowing p38 to be induced much faster. Prior to determining the other mechanisms working with p38, however, we wanted to confirm that p38 was indeed causing receptor changes by evaluating the number of REDV binding receptors following p38 inhibition (Fig. 4). This was achieved by first inhibiting p38 followed by incubation in the mentioned concentrations of REDV. These cells were either subjected to shear stress (dynamic) following p38 inhibition or not (static). Fluorescently labeled REDV-FITC was allowed to bind to the unoccupied REDV sites. The results from this experiment were compared to the uninhibited state represented in Fig. 1. The inhibition of p38 does not interfere with the existing receptors as reflected by the presence of similar numbers of receptors for the inhibited and uninhibited cells that have not been incubated in REDV (0 µg/mL REDV; all four cases) (Fig. 4). The addition of REDV to the inhibited cells results in a decrease in unoccupied REDV receptors. This trend is observed in both the static and dynamic cases. This result is consistent with the observations made in the adhesion based inhibition studies which suggested that p38 was responsible for the rapid receptor number increase. In general, the intracellular localization of p38 following stimulation of cells by shear or other stressors is not well understood [37]; consequently we sought to determine the mechanism by which it is activated in the context of this study.

The ability of cells to internalize and recycle receptors in a short time period (2 min or less) is well known [38], [39], [40]. Such changes in surface receptor abundance have, for example, been implicated in the observed decrease in efficacy of antibody-based therapeutics [41]. The other potential mechanism for cells to become more adhesive is via new receptor synthesis. However, such synthesis is known to require at least 6 hours for the RNA transcription and translation of the new receptor [42], [43], a time frame that is well outside the range of our experiments.

**Figure 5:**
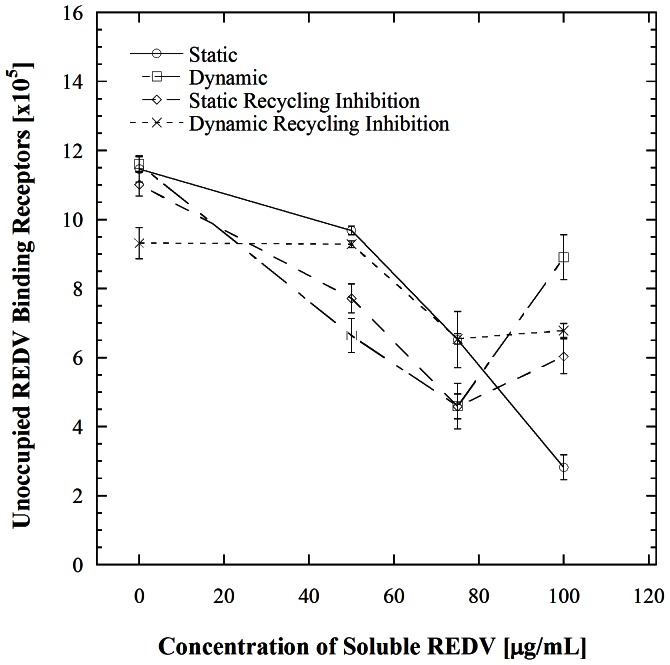
Receptor number analysis after the inhibition of receptor recycling followed by incubation in soluble REDV and comparison with and without shear exposure via flow through REDV-coated microchannels. Error bars denote standard errors for each point based on 3 repetitions. Upon pre-incubation in100 µg/mL of REDV without recycling inhibition, the static and dynamic cases are not statistically different (*p* = 0.161), indicating that recycling plays a role in the rapid presentation of receptors observed in the uninhibited condition. Statistical comparisons 50, 75, and 100 µg/mL points within each condition are provided as Supplementary Information.

We hypothesized that if the REDV-binding receptors on HUVECs were undergoing internalization or recycling, such changes would be observable via cell adhesion experiments. Hence the next series of experiments examined inhibition of receptor recycling. Fig. 5 shows the results from this experiment wherein the number of unoccupied REDV receptors was determined. The inhibition of receptor recycling followed by REDV incubation only, results in a decrease in receptor number as the concentration of REDV increases. This result suggests that incubation in REDV alone does not cause receptors to be internalized or recycled. When these inhibited cells are also flowed into REDV-coated microchannels there is also a decrease in receptor numbers even at the highest concentration of REDV (dynamic receptor recycling inhibition). This result is noteworthy because it suggests that dynamic receptor recycling inhibition suppresses the surface presentation of additional receptors that otherwise become available for binding with REDV at the condition of 100 µg/mL soluble REDV plus shear exposure. It is also interesting to observe that receptor recycling inhibition appears to mitigate the effect of shear exposure. In other words, at the condition of 100 µg/mL soluble REDV (in Fig. 5), where the greatest difference in the number of REDV receptors is observed in the absence of recycling inhibition, such inhibition eliminates the distinction between the static and dynamic cases but nonetheless allows a greater number of REDV receptors to be present at the cell surface (relative to the uninhibited static condition). On one hand, this observation points to the essential role that the soluble REDV plays in increasing the number of receptors via recycling; however, importantly, this observation also establishes a connection between receptor recycling and shear exposure.

**Figure 6:**
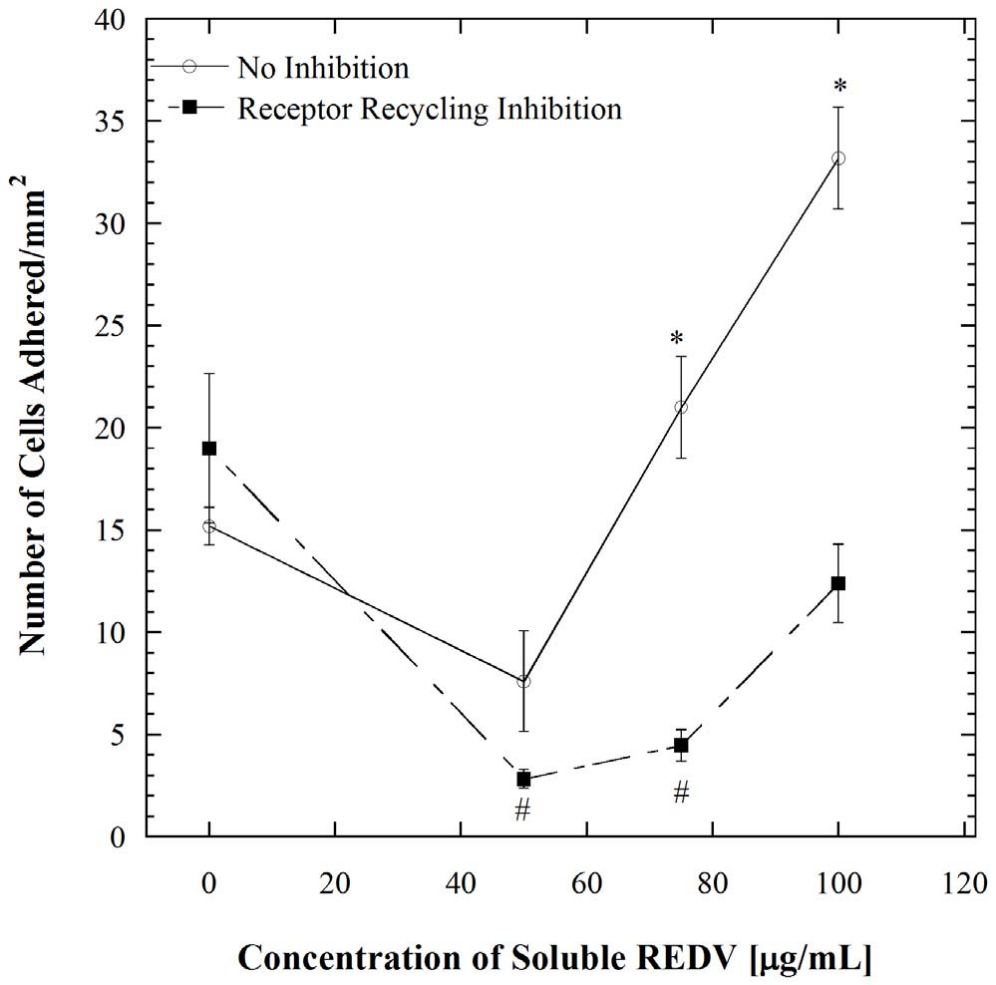
Adhesion of HUVECs in REDV-coated channels at a shear stress of 1.1 dyn/cm^2^ following receptor recycling inhibition and incubation with various concentrations of REDV. Error bars denote standard errors for each point based on 5 repetitions. * denotes significant difference with *p*<0.001 compared to the no inhibition condition for each REDV concentration. A comparison of cell adhesion between cells pre-incubated in 75 and 100 µg/mL of REDV following recycling inhibition shows no significant difference between the two conditions (*p = 0.005*) even though cell adhesion increases at the 100 µg/mL region.

The receptor recycling studies were further validated with cell adhesion experiments and the results are shown in Fig. 6. HUVECs were inhibited of receptor recycling followed by incubation in the various concentrations of REDV and flow in REDV-coated microchannels and adhered cells were enumerated. (This series of experiments focuses on the ‘dynamic’ case.) The cell adhesion data is consistent with the receptor number observations. Overall, the inhibition of receptor recycling results in significantly lower cell adhesion for all levels of soluble REDV examined, but nevertheless retaining the trend of increased adhesion as soluble REDV concentration is increased. These data conclusively show that receptor recycling is indeed occurring under the conditions examined and to a significant extent.

**Figure 7:**
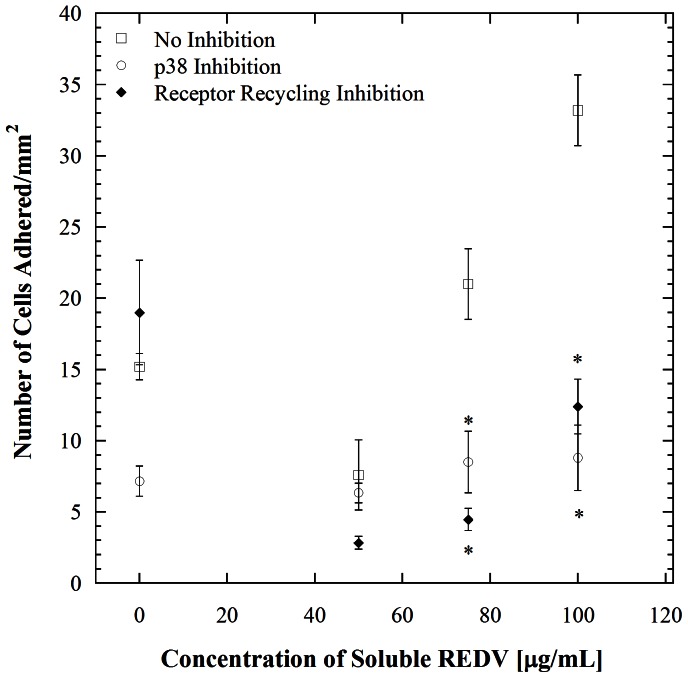
Comparison of p38 and receptor recycling inhibitory effects on cell adhesion to REDV-coated channels at a shear stress of 1.1 dyn/cm^2^. Error bars denote standard errors for each point based on 5 repetitions. *denotes significant difference with *p*<0.001 compared to the no inhibition condition for each REDV concentration.

Several major insights are provided by the studies described above. From a mechanistic standpoint, it is now clear that the enhancement in HUVEC adhesion observed in our earlier study [15] resulting from incubation with soluble REDV and shear exposure is a result of p38 pathway-mediated receptor recycling. Signaling by downstream kinases is known to affect endocytosis and endosomal trafficking at many points [44]. The stress-induced p38 kinase stimulates the formation of RAB5 and GDI complexes that consequently causes accelerated endocytosis. Studies of this effect in the context of epidermal growth factor receptor (EGFR) have shown that activation of p38 promotes the internalization of EGFR that is not bound to a ligand [45], [46]. Further, such endocytosis is subcategorized into early and late endocytosis [47]. In early endocytosis, internalized molecules are either recycled back to the plasma membrane or delivered to the next step of the endocytic pathway, also referred to as the late endosome, where lysosome-mediated degradation occurs [46]. In our work, it is apparent that p38 induces such early endocytosis, which is followed by reintroduction of the receptors to the cell surface. This is evident in Figure 7 where cell adhesion trends between p38 and receptor recycling inhibition are compared to the non inhibited case. There is a significant decrease in cell adhesion when cells are inhibited of either p38 or receptor recycling (*p*<0.001), while no significant difference exists between the two inhibited cases (*p*>0.001). Our results also show an absence in ERK 1/2 induction, which is consistent with literature showing that ERK 1/2 recycles receptors at a much later stage [46], [47], [48], [49], [50]. Indeed, it is noteworthy that the relatively simple cell adhesion assays described herein were able to recapitulate the link between p38 and receptor surface presentation. The experimental platform employed herein however does not allow independent assessments of receptor engagement with soluble versus insoluble (immobilized) ligands. Such engagement mechanisms are highly context-dependent; for example an instance where a cell has equal opportunity to interact with soluble and insoluble ligand in terms of physical proximity and time would be very different from one where such interactions occur separately and along different time scales. This difference would be manifested in a variety of different ways, ranging from signaling pathway activation to differences in cell adhesiveness.

Our studies demonstrate how intracellular events can impact adhesiveness of cells, a key consideration in the design of cell separation systems based on ligand-receptor affinity [14], [51]. A major distinction between this study and the majority of studies in the area of shear-mediated intracellular events is that the cells are not previously plated and adhered to a surface prior to shear exposure [52], [53], and a very simple cell adhesion assay platform is utilized to probe intracellular events. More broadly, the methodology utilized herein, specifically a simple combination of soluble ligand, recycling inhibitor and a linear microfluidic channel, can be applied to screen combinations of cells, soluble factors, immobilized ligands for receptor recycling phenomena. Such studies may enhance the process of drug discovery by being able to characterize and identify conditions that can result in drug target receptor endocytosis. While the present manuscript demonstrates the capabilities of our simple platform, a more complete mechanistic understanding would require the incorporation of appropriate knockdowns. For example, HUVECs with p38 knocked down would shed insights into the extent that p38 induces the receptor recycling observed [54].

## Conclusion

We demonstrate the surface presentation of REDV receptors in HUVECs is affected by both mitogen-activated protein kinases and receptor recycling as reflected by inhibition studies that measured cell adhesion and cell receptor number changes. The changes observed in REDV receptor number and the associated changes in adhesiveness are relevant not only to the design of affinity-based cell separation methodologies but also reflect how soluble factor- and shear-mediated intracellular signalling and receptor recycling events can be monitored via a relatively simple cell adhesion assay.
